# Factors predicting long-term outcomes of early-stage hepatocellular carcinoma after primary curative treatment: the role of surgical or nonsurgical methods

**DOI:** 10.1186/s12885-021-07948-9

**Published:** 2021-03-08

**Authors:** Ming-Jeng Kuo, Lein-Ray Mo, Chi-Ling Chen

**Affiliations:** 1 Department of Hepatogastroenterology, Tainan Municipal Hospital (Managed by Show Chwan Medical Care Corporation), Tainan 701, Taiwan. No. 670, Chon-De Road, Tainan, 701 Taiwan; 2 Graduate Institute of Clinical Medicine, College of Medicine, and Graduate Institute of Epidemiology and Preventive Medicine, College of Public Health, National Taiwan University, Taipei 100, 7 Chung-Shan South Road, Taipei, 100 Taiwan

**Keywords:** Early hepatocellular carcinoma, Radiofrequency ablation, Transcatheter arterial chemoembolization, Surgical resection, Prognosis, Propensity score matching

## Abstract

**Background:**

We quantified the elusive effects of putative factors on the clinical course of early hepatocellular carcinoma (HCC) after primary surgical or nonsurgical curative treatment.

**Methods:**

Patients with newly diagnosed early HCC who received surgical resection (SR) or percutaneous radiofrequency ablation (RFA) with or without transcatheter arterial chemoembolization (TACE) from January 2003 to December 2016 were enrolled. The cumulative overall survival (OS) and disease-free survival (DFS) rates were compared. A polytomous logistic regression was used to estimate factors for early and late recurrence. Independent predictors of OS were identified using Cox proportional hazard regression.

**Results:**

One hundred twenty-five patients underwent SR, and 176 patients underwent RFA, of whom 72 were treated with TACE followed by RFA. Neither match analysis based on propensity score nor multiple adjustment regression yielded a significant difference in DFS and OS between the two groups. Multivariate analysis showed high AFP (> 20 ng/mL), and multinodularity significantly increased risk of early recurrence (< 1 year). In contrast, hepatitis B virus, hepatitis C virus and multinodularity were significantly associated with late recurrence (> 1 year). Multivariate Cox regression with recurrent events as time-varying covariates identified older age (HR = 1.55, 95% CI:1.01–2.36), clinically significant portal hypertension (CSPH) (HR = 1.97, 95% CI:1.26–3.08), early recurrence (HR = 6.62, 95% CI:3.79–11.6) and late recurrence (HR = 3.75, 95% CI:1.99–7.08) as independent risk factors of mortality. A simple risk score showed fair calibration and discrimination in early HCC patients after primary curative treatment. In the Barcelona Clinic Liver Cancer (BCLC) stage A subgroup, SR significantly improved DFS compared to RFA with or without TACE.

**Conclusion:**

Host and tumor factors rather than the initial treatment modalities determine the outcomes of early HCC after primary curative treatment. Statistical models based on recurrence types can predict early HCC prognosis but further external validation is necessary.

**Supplementary Information:**

The online version contains supplementary material available at 10.1186/s12885-021-07948-9.

## Introduction

Hepatocellular carcinoma (HCC) is one of the most common cancers and one of the leading causes of malignancy-related death worldwide [1]. Since the launch of routine ultrasound and alpha-fetoprotein surveillance in high-risk populations, more and more patients are being diagnosed with HCC at an early stage, which is beneficial to curative therapies [2, 3]. Because of the shortage of donor organs, surgical resection (SR) and nonsurgical methods, including radiofrequency ablation (RFA) alone or the combined use of transcatheter arterial chemoembolization (TACE) remain the mainstay of curative HCC treatment in Asian-Pacific countries [4].

For patients with early HCC, SR has been proved to provide better clinical outcomes than does local ablation, though said better outcomes were limited to well-preserved liver function [5, 6]. However, RFA has begun to challenge the status of SR as the optimal treatment for early HCC < 2 cm in terms of sustained local tumor control and survival [7]. Currently, the combined use of transcatheter arterial chemoembolization (TACE) and RFA has broadened this challenge, being widely accepted as the preferred strategy for intermediate-sized (3.1–5.0 cm) HCC treatment [8]. Given the influence of various tumor and liver reserve factors, the choice of either a RFA with or without TACE (RFA-TACE) method or a SR method is of great interest to clinical physicians in the management of early-stage HCC.

Recurrence after curative treatment remains a big challenge for clinical physicians. Intrahepatic metastasis and multicentric HCC developed through the accumulation of genetic alternations were previously thought to be major mechanisms for early and late HCC recurrence, respectively [9, 10]. Identification of patients who are at risk of recurrence after curative treatment allows clinicians to provide intensive surveillance and detect recurrent tumors at earlier stages, when curative treatment is still feasible. In addition, a few models from both eastern and western countries have been developed specifically to predict the long-term survival rate after curative HCC management, but none of them have taken the influence of different recurrence types into consideration [11, 12].

The aim of our study is to determine if the initial treatment modalities or other clinical factors that could predict recurrence and overall survival rates of early HCC after primary curative treatment. A simple scoring system is also established for HCC outcome predictions.

## Patients and methods

This is a cohort study conducted as a retrospective analysis of a prospective database at a single institution. The study cohort consists of patients who were newly diagnosed HCC from January 2003 to December 2016. The inclusion criteria were (1) a single HCC <50 mm or up to 3 HCCs <30 mm, (2) no previous treatment for HCC, (3) Child-Pugh class A or B cirrhosis, and (4) no vascular invasion or extrahepatic metastasis. Patients with an Eastern Cooperative Oncology Group performance status of 2 or greater, and those with the presence of an uncontrollable malignancy other than HCC were excluded. The diagnosis of HCC was made using European Association for the Study of the Liver (EASL) and American Association for the Study of Liver Diseases (AASLD) guidelines [13, 14]. The study protocol was approved by the ethics committee of Show Chwan Memorial Hospital.

### HCC management

The surgical resection (SR) comprised the mainstay of surgical method and was determined according to tumor location, the extent of the tumor, hepatic reserve function, and patients’ general condition. Liver reserve was assessed by Child-Pugh classification and indocyanine green retention rate at 15 minutes. The extent of SR was mainly based on the algorithm proposed by Makuuchi et al. and anatomic resection was performed if the liver function was fair [15]. The nonsurgical method included both RFA and sequential RFA after TACE treatment. All RFA procedures were performed percutaneously under general anesthesia. Real-time ultrasound was chosen as the guidance modality. RFA was done by two senior gastroenterologists with at least 10 years of experience. The RFA was performed with one of the following devices: monopolar expandable Boston LeVeen™ needles (RF 3000 Boston Scientific Corporate^®^); Cool-tip™ RFA system (Covidan^®^); Dual- Switching Systems (VIVA Multi^®^); or Separable clustered electrode (Octopus).

TACE was usually performed prior to the RFA procedures for patients with HCC near large vessels, solitary intermediate-sized (3.1-5.0 cm in maximum diameter) or multinodular HCC, and relatively preserved liver function (mostly Child-Pugh A or B7 without ascites). Except for a few cases in which drug-eluting beads were used, most of our patients received conventional TACE. The procedure was performed via intra-arterial injection of a viscous emulsion which consists of doxorubicin mixed with lipiodol, followed by embolization of the blood vessel with gelatin sponge particles. Few of our subjects underwent liver transplantation, not only because the organ resources were short in Taiwan, but also because this therapeutic option was only available in limited centers.

### Data collection and follow-up

The main variable of interest was the type of HCC recurrence. Tumor recurrence was classified as no, early phase, and late phase by using 12 months as cutoff. The demographic and clinicopathological information of all the participants was collected via retrospective chart review by physicians. Subjects who were on antihyperglycaemic treatment were considered to be diabetics. Clinically significant portal hypertension (CSPH) was diagnosed if one of the following criteria was met: 1) the patient had esophageal or gastric varices confirmed by endoscopy or 2) splenomegaly on imaging and a platelet count less than 100,000/uL. Major complications were defined as those that led to prolonged hospital stay, hospital admission or additional necessitated therapy.

Patients were assessed by serum biochemistry and ultrasonography every 3 months and by computed tomography scan or magnetic resonance imaging every 6 months after curative RFA or SR. Once recurrence was found, patients were managed with either SR, RFA or TACE. The duration of follow-up was recorded from the day of curative management until loss to follow-up, death or Dec 31, 2017.

### Statistical analysis

Chi-square tests and Student’s t-tests were used to compare the differences between the two groups with regard to clinical characteristics. The Nelson-Aalen cumulative hazard estimate and the log-rank test were used to compare the OS and DFS rates of the HCC patients treated with SR or nonsurgical methods. The propensity score was calculated by a multivariate logistic regression model which allows users to save the predicted probability of each patient being assigned to each option of curative treatment. Variables involving the recurrence or survival of HCC were entered in the propensity score model. One-to-one matching of propensity score was used to balance the baseline differences between the nonsurgical and SR groups. The difference between matched pairs was evaluated using signed rank test for continuous data and McNemar’s test for binary data.

Polytomous logistic regression was used to assess independent risk factors for early or late recurrence of early HCC after curative treatment. Variables with values of *p* < 0.1 in the univariate analysis were further included in the multivariate regression analysis. Cox proportional hazards regression models were conducted to estimate the clinicopathologic factors associated with long-term survival. To establish a multivariate predicted model, we used forward selection with *p* < 0.15 to evaluate the additive effects of risk factors. The final model was selected on the basis of log-likelihood test and Akaike information criterion. By using the set of variables that were significant in the final model, a predicted risk score composed of time-invariant and time-varying factors based on the regression coefficients estimated from the final model was developed. After we excluded enrolling subjects who did not complete 3 or 5 years of follow-up with censored observations, the discrimination capabilities were presented by receiver operating characteristic (ROC) curve and the optimal cut-off was estimated by using Youden index. All analysis was conducted with SAS version 9.4. All statistical tests were 2-sided and *p* < 0.05 indicated significance.

## Results

### Patients

A total of 312 patients who underwent curative HCC management with pre-treatment serum and post-treatment pathology-verified HCC samples were collected as the target population. After excluding those with either residual tumors after nonsurgical therapy or those without free margin after SR, 301 patients who received SR, TACE followed by RFA, or RFA alone as the initial curative treatment for HCC were enrolled. One hundred twenty-five patients underwent SR. The operative procedure consisted of partial resections in 43 (34.4%) of those cases, segmentectomies/bisegmentectomies in 66 (52.8%) cases, and trisegmentectomy/lobectomy in 16 (12.8%) cases. On the other hand, 176 patients underwent RFA, of whom 72 (41%) were treated with TACE followed by RFA. In our RFA-TACE cohort, 88% (29/33) of the patients with Barcelona Clinic Liver Cancer (BCLC) very early stage HCC received RFA monotherapy. In addition, more than 60 % (49/81) of the patients with either multinodularity or a single tumor of more than 3 cm in size underwent combined therapy (that is, TACE followed by RFA) for control of early HCC. The median follow-up times of the RFA-TACE (that is, those treated with RFA alone or with TACE followed by RFA) and SR groups were 32.2 months and 33.8 months, respectively.

### Comparison of baseline characteristics of RFA-TACE and SR groups before and after propensity score matching

A comparison of all patients in our original cohort before propensity score matching revealed that there were no significant differences in gender, age, Ishak score, Edmonson grading, history of diabetes, CSPH, levels of total bilirubin, alanine aminotransferase, aspartate aminotransferase, total serum albumin and renal function impairment. However, the RFA-TACE group did include a larger proportion of patients with chronic hepatitis C virus (HCV) infection. With regard to liver reserve factors, the SR patients were significantly more likely to have well-preserved liver function (Child-Pugh class A) (*P* = 0.04). On the other hand, with regard to tumor factors, the patients who underwent RFA-TACE had higher levels of serum AFP and a larger proportion of multinodularity and Barcelona Clinic Liver Cancer (BCLC) stage 0. However, the SR patients had larger tumor sizes in comparison to the RFA-TACE patients (*P* < 0.0001). The baseline characteristics of the RFA-TACE and SR groups are listed in Table 1. Through propensity score matching, 66 matching pairs were generated. The confounding variables contributing to treatment selection were well matched and no significant differences were found between the SR and RFA-TACE groups (Table 1).

**Table 1:** Clinical characteristics of early hepatocellular carcinoma patients treated with RFA-TACE or with SR before and after propensity score matching

Variables	Before propensity score matching			After propensity score matching		
	RFA-TACE (*n* = 176)	SR (*n* = 125)	*P* value	RFA-TACE (*n* = 66)	SR (*n* = 66)	*P* value
	No. (%)	No. (%)		No. (%)	No. (%)	
Age (years)						
< 65	90 (51.1)	74 (59.2)	0.17	35 (53.0)	35 (53.0)	1.0
> 65	86 (48.9)	51 (40.8)		31 (47.0)	31 (47.0)	
Mean + SD	64.0 + 10.4	62.2 + 10.7		63.7 + 12.6	64.0 + 10.3	
Gender						
Male	109 (61.9)	87 (69.6)	0.17	47 (68.1)	47 (68.1)	1.0
Female	67 (38.1)	38 (30.4)		22 (31.9)	22 (31.9)	
Child-Pugh						
A	162 (92.1)	122 (97.6)	0.04	61 (92.4)	65( 98.5)	0.10
B	14 (7.9)	3 (2.4)		5 (7.6)	1 (1.5)	
Etiology						
Seronegative	17 (9.7)	21 (16.8)	0.008	8 (12.1)	7 (10.6)	0.40
HBV	70 (39.8)	65 (52.0)		34 (51.5)	28 (42.4)	
HCV	82 (46.6)	36 (28.8)		22 (33.3)	29 (43.9)	
HBV + HCV	7 (4.0)	3 (2.4)		2 (3.03)	2 (3.03)	
Ishak score-fibrosis						
1–3	52 (29.6)	29 (23.2)	0.22	16 (24.2)	14 (21.2)	0.67
4–6	124 (70.4)	96 (76.8)		50 (75.8)	52 (78.8)	
Edmonson grading						
I and II	137 (90.7)	103 (83.1)	0.06	50 (89.3)	48 (85.7)	0.56
III and IV	14 (9.3)	21 (16.9)		6 (10.7)	8 (14.3)	
Diabetes						
No	123 (69.9)	92 (73.6)	0.48	47 (71.2)	48 (72.7)	0.85
Yes	53 (30.1)	33 (26.4)		19 (28.8)	18 (27.3)	
CSPH						
No	131 (74.4)	99 (79.2)	0.34	55 (79.7)	53 (76.8)	0.69
Yes	45 (25.6)	26 (20.8)		14 (20.3)	16 (23.2)	
No. of nodules						
Single	126 (71.6)	104 (83.2)	0.02	58 (87.9)	56 (84.9)	0.56
> 2 nodules	50 (28.4)	21 (16.8)		8 (12.1)	10 (15.1)	
Creatinine (mg/dL)						
< 1.5	159 (90.3)	120 (96.0)	0.07	62 (93.9)	64 (97.0)	0.41
> 1.5	17 (9.7)	5 (4.00)		4 (6.1)	2 (3.0)	
AFP level (ng/mL)						
< 20	96 (54.6)	85 (68.0)	0.02	40 (60.6)	42 (63.6)	0.59
> 20	80 (45.4)	40 (32.0)		26 (39.4)	24 (36.4)	
Albumin (mg/dL)						
> 3.0	166 (94.3)	123 (98.4)	0.07	61 (92.4)	65 (98.5)	0.05
< 3.0	10 (5.7)	2 (1.6)		5 (7.6)	1 (1.5)	
Total bilirubin (mg/dL)						
< 1.5	161 (91.5)	117 (95.1)	0.22	60 (90.9)	61 (92.4)	0.74
> 1.5	15 (8.5)	6 (4.9)		6 (9.1)	5 (7.6)	
Prothrombin time (INR)						
< 1.3	164 (93.2)	122 (97.6)	0.11	62 (93.9)	65 (98.5)	0.18
> 1.3	12 (6.8)	3 (2.4)		4 (6.1)	1 (1.5)	
AST (IU/L)						
< 80	139 (79.0)	106 (84.8)	0.20	56 (84.9)	55 (83.3)	0.81
> 80	37 (21.0)	19 (15.2)		10 (15.1)	11 (16.7)	
ALT (IU/L)						
< 80	140 (79.6)	99 (79.2)	0.94	54 (81.8)	53 (80.3)	0.81
> 80	36 (20.5)	26 (20.8)		12 (18.2)	13 (19.7)	
Size (cm)						
< 3.0	129 (73.3)	50 (40.0)	< 0.0001	38 (57.6)	37 (56.1)	0.76
> 3.0	47 (26.7)	75 (60.0)		28 (42.4)	29 (43.9)	
Mean + SD	24.9 + 9.0	32.6 + 11.5		27.5 + 10.2	28.3 + 10.8	
BCLC stage						
0	47 (26.7)	18 (14.4)	0.01	17 (25.8)	16 (24.2)	0.71
A	129 (73.3)	109 (85.6)		49 (74.2)	50 (75.8)	

*CSPH* clinically significant portal hypertension, *AFP* Alpha-fetoprotein, *ALT* Alanine aminotransferase, *AST* Aspartate aminotransferase, *HBV* hepatitis B virus, *HCV* hepatitis C virus, *INR* International normalized ratio, *BCLC* Barcelona-Clınic Liver Cancer staging system, *RFA-TACE* Radiofrequency ablation with or without transcatheter arterial chemoembolization, *SR* surgical resection

### Survival analysis in both groups

**Fig. 1:**
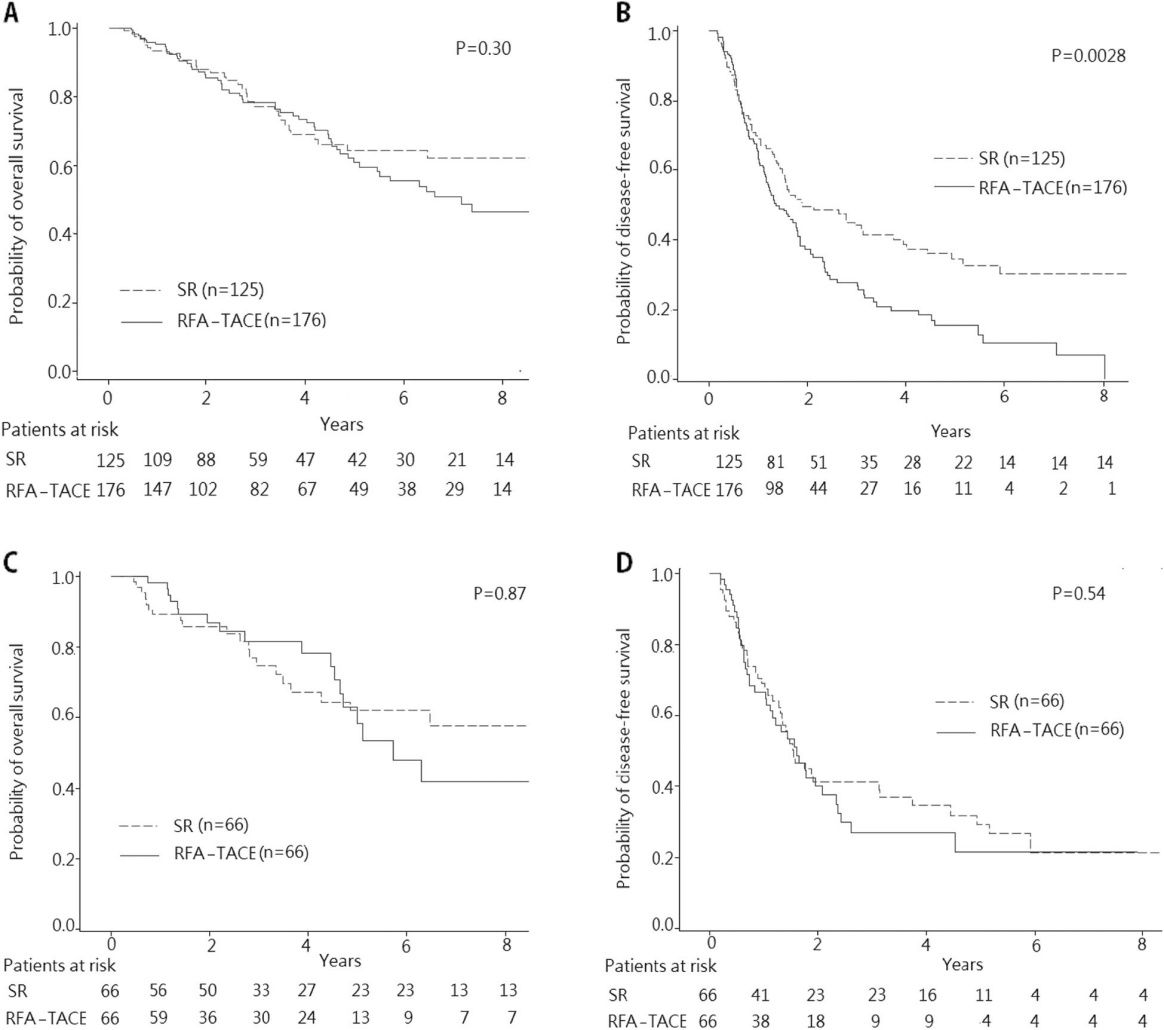
Comparison of survival curves of the patients with early-stage HCC who underwent RFA-TACE or SR. **a** Cumulative OS curves of patients who underwent RFA-TACE and patients who underwent SR. **b** Cumulative DFS curves of patients who underwent RFA-TACE and patients who underwent SR. **c** The cumulative OS curves of patients who underwent RFA-TACE and patients who underwent SR after propensity score matching. **d** The cumulative DFS curves of patients who underwent RFA-TACE and patients who underwent SR after propensity score matching

Figure 1 illustrates the OS and DFS rates of the SR and RFA-TACE groups. In the RFA-TACE group, the 1-, 3-, and 5-year cumulative OS rates were 95.2, 78.4, and 60.9%, respectively, while the 1-, 3-, and 5-year DFS rates were 66.2, 28.0 and 15.7%, respectively. On the other hand, the 1-, 3-, and 5-year cumulative OS rates of the SR group were 93.4, 77.2, and 64.5%, respectively, while the 1-, 3-, and 5-year DFS rates were 68.9, 43.9, and 34.4%, respectively. There were no significant differences in OS rates between the two groups (*P* = 0.30) (Fig. 1a). However, in comparison with the patients who underwent SR, the patients who underwent RFA-TACE had significantly more recurrence (*P* = 0.0028) (Fig. 1b).

After propensity score matching, the 1-, 3-, and 5-year OS rates of the RFA-TACE group were 98.3, 81.5, and 58.2%, respectively, compared to 1-, 3-, and 5-year OS rates of 89.0, 74.7, and 61.9%, respectively, for the SR group. There were thus no statistically significant differences in term of OS between the patients receiving RFA-TACE and SR (*P* = 0.87) (Fig. 1c). Similar results were obtained between the two groups in terms of recurrence. The 1-, 3-, and 5-year DFS rates of the RFA-TACE group were 66.4, 27.1, and 21.7%, respectively, compared to 1-, 3-, and 5-year DFS rates of 67.3, 41.3, and 29.4%, respectively, for the SR group (*P* = 0.54) (Fig. 1d). Concerning the clinical course from recurrence to death, there were no statistical differences between RFA-TACE and SR groups in terms of post-recurrence survival (*P* = 0.43).

### Complications

There was no mortality during the initial hospital stays for either group. Two major complications (1.1%) occurred in two patients after RFA therapy. Specifically, one patient experienced intraperitoneal bleeding that required a blood transfusion and subsequent transcatheter arterial embolization, while the other patient had hemobilia, such that an endoscopic sphinterotomy for the removal of blood clots was required. Three major complications (2.4%) were recorded after SR. One patient developed a postoperative abscess that required surgical debridement, while liver decompensation including jaundice, ascites, and encephalopathy occurred in two patients. There was no significant difference between the major complication rates of the two groups (*P* = 0.65).

### Predictors for HCC recurrence

Table 2 shows the results for the one-by-one testing of covariates for HCC early and late recurrence. It was found that CSPH, advanced fibrosis (FIB-4 > 3.25), multinodularity, higher AFP (> 20 ng/mL) and HCV raised the likelihood of early recurrence. In contrast, elevated alanine aminotransferase (ALT), CSPH, advanced fibrosis, multinodularity, hepatitis B virus (HBV) and HCV contributed to the development of late recurrence. In the multivariate analysis, multinodularity (OR = 3.66, 95% CI = 1.72–7.80) and higher AFP (OR = 2.06, 95% CI = 1.12–3.81) were the factors that contributed significantly to early recurrence. On the other hand, multinodularity (OR = 2.50, 95% CI = 1.14–5.50), chronic HBV infection (OR = 5.11, 95% CI = 1.59–16.4) and chronic HCV infection (OR = 8.07, 95% CI = 2.41–27.0) were associated with a significantly increased risk of late recurrence (Table 2).

**Table 2:** Predictors for early and late recurrence after primary curative treatment by using polytomous logistic regression

Variables	Univariate model (OR, 95% CI)		Multivariate model (OR, 95% CI)	
	Early recurrence (< 1 year) vs No	Late recurrence (> 1 year) vs No	Early recurrence (< 1 year) vs No	Late recurrence (> 1 year) vs No
Host-related factors				
Age > 65 years	1.08(0.62–1.89)	1.31 (0.76–2.27)		
Male	1.26 (0.70–2.27)	1.57 (0.88–2.79)		
AST > 80 IU/L	1.49 (0.72–3.09)	1.62 (0.79–3.33)		
ALT > 80 IU/L	1.59 (0.77–3.28)	2.15 (1.07–4.31)		
Total bilirubin > 1.5 mg/dL	0.81 (0.29–2.21)	0.43 (0.13–1.41)		
Prothrombin time (INR) > 1.3	0.69 (0.16–2.98)	1.63 (0.50–5.32)		
Albumin <3.0 mg/dL	1.69 (0.52–5.51)	0.90 (0.24–3.46)		
Child-Pugh score B (vs A)	1.39 (0.45–4.30)	0.75 (0.20–2.72)		
CSPH	2.97 (1.48–5.96)	2.32 (1.15–4.70)	2.03 (0.88–4.67)	1.63 (0.71–3.75)
Creatinine > 1.5 mg/dL	1.19 (0.43–3.29)	0.84 (0.28–2.51)		
FIB-4 index > 3.25	2.15 (1.22–3.79)	1.86 (1.06–3.25)	1.40 (0.67–2.90)	1.13 (0.56–2.30)
Diabetes	1.37 (0.74–2.54)	1.38 (0.75–2.54)		
ALBI grade 2 or 3 (vs 1)	1.29 (0.63–2.61)	1.17 (0.59–2.34)		
Tumor characteristics				
Size > 3 cm	0.84 (0.48–1.47)	0.80 (0.46–1.39)		
Multinodularity	3.71 (1.84–7.49)	2.25 (1.09–4.66)	3.66 (1.72–7.80)	2.50 (1.14–5.50)
AFP > 20 ng/mL	2.33 (1.31–4.14)	1.64 (0.92–2.91)	2.06 (1.12–3.81)	1.40 (0.75–2.58)
BCLC stage A (vs 0)	1.55 (0.79–3.05)	1.32 (0.68–2.53)		
Histopathological findings				
Edmonson grade III & IV	0.96 (0.36–2.56)	2.04 (0.88–4.72)		
Ishak fibrosis score 4–6	0.94 (0.50–1.74)	0.98 (0.53–1.81)		
Viral factors				
HBV	1.61 (0.70–3.68)	4.75 (1.53–14.8)	1.88 (0.77–4.63)	5.11 (1.59–16.4)
HCV	2.63 (1.11–6.21)	8.90 (2.81–28.2)	2.44 (0.93–6.41)	8.07 (2.41–27.0)
HBV and HCV	2.09 (0.44–9.96)	2.87 (0.39–21.3)	1.13 (0.19–6.58)	1.91 (0.24–15.5)
Treatment modality				
RFA-TACE (vs Surgery)	1.32 (0.72–2.30)	1.59 (0.91–2.78)		

*aHR* adjusted hazard ratio, *ALT* alanine aminotransferase, *AST* aspartate aminotransferase, *INR* international normalized ratio, *CSPH* clinically significant portal hypertension, *ALBI* albumin-bilirubin, *AFP* alpha-fetoprotein, *HBV* hepatitis B virus, *HCV* hepatitis C virus, *BCLC* Barcelona-Clınic Liver Cancer staging system, *RFA-TACE* Radiofrequency ablation with or without transcatheter arterial chemoembolization

### Predictors for overall survival and derivation of predicted score

Univariate analysis by Cox regression revealed that the overall survival was significantly associated with early recurrence (< 1 year), late recurrence (> 1 year), older age (> 65 years), hypoalbuminemia (< 3.0 mg/dL), CSPH, ALBI grade 2 or 3, FIB-4 index> 3.25, BCLC stage A and chronic viral hepatitis (Table 3). We further used forward selection to evaluate the additive effects of covariates on overall survival. Model I included the potential predictors without recurrence. Early and late recurrence were considered time-invariant and time-varying covariates in model II and model III, respectively. On the basis of log-likelihood ratio and Akaike information criterion tests, the final model included early recurrence, late recurrence, older age and clinically significant portal hypertension (CSPH) (Table 3).

**Table 3:** Predictors for overall survival after primary curative treatment by using Cox regression model

Variables	Univariate model	^a^Multivariate model		
	HR (95% CI)	Model I Time-invariant without recurrence aHR (95% CI)	Model II Time-invariant with recurrence aHR (95% CI)	Model III Time-varying with recurrence aHR (95% CI)
**Time-dependent variables**				
Recurrence type				
No	1.00			1.00
Early recurrence (< 1 year)	9.44 (4.98–17.9)			6.62 (3.79–11.6)
Late recurrence (> 1 year)	6.46 (3.17–13.2)			3.75 (1.99–7.08)
**Time-independent variables**				
Recurrence type				
No	1.00		1.00	
Early (< 1 year)	4.51 (2.42–8.39)		3.09 (2.03–4.71)	
Late (> 1 year)	1.73 (0.91–3.28)			
Host-related factors				
Age > 65 years	1.64 (1.09–2.48)		1.43 (0.92–2.21)	1.55 (1.01–2.36)
Male	1.23 (0.81–1.86)			
AST > 80 IU/L	1.30 (0.80–2.11)			
ALT > 80 IU/L	0.99 (0.60–1.63)			
Total bilirubin > 1.5 mg/dL	1.93 (1.00–3.72)			
Prothrombin time (INR) > 1.3	1.86 (0.86–4.04)			
Albumin <3.0 mg/dL	2.38 (1.20–4.75)			
Child-Pugh score B (vs A)	1.63 (0.75–3.53)			
CSPH	2.31 (1.50–3.56)	1.53 (0.93–2.52)	1.71 (1.04–2.82)	1.97 (1.26–3.08)
Creatinine > 1.5 mg/dL	2.04 (0.98–4.22)	1.93 (0.92–4.02)	1.89 (0.90–3.97)	
Diabetes	1.23 (0.79–1.90)			
ALBI grade 2 or 3 (vs 1)	1.91 (1.02–3.59)			
FIB-4 index > 3.25	2.50 (1.63–3.84)	2.02 (1.23–3.31)	1.83 (1.12–3.01)	
Tumor characteristics				
Size > 3 cm	1.02 (0.67–1.54)			
Multinodularity	1.18 (0.75–1.84)			
AFP > 20 ng/mL	1.20 (0.79–1.81)			
BCLC stage A (vs 0)	1.94 (1.03–3.65)	1.88 (1.00–3.55)		
Histopathological findings				
Edmonson grade III & IV	1.35 (0.76–2.40)			
Ishak fibrosis score 4–6	1.24 (0.79–1.96)			
Viral factors				
HBV	5.23 (2.86–9.57)			
HCV	5.36 (2.96–9.70)			
HBV and HCV	10.3 (3.77–28.1)			
Treatment modality				
RFA-TACE (vs. Surgery)	1.25 (0.82–1.91)			
**Model selection**				
-2 log likelihood ratio		871.9	847.7	834.5
AIC		879.9	857.7	842.5

*aHR* adjusted hazard ratio, *ALT* alanine aminotransferase, *AST* aspartate aminotransferase, *INR* international normalized ratio, *CSPH* clinically significant portal hypertension, *ALBI* albumin-bilirubin, *AFP* alpha-fetoprotein, *HBV* hepatitis B virus, *HCV* hepatitis C virus, *BCLC* Barcelona-Clınic Liver Cancer staging system, *RFA* radiofrequency ablation, *AIC* Akaike information criterion

^a^Multivariate analyses were performed by the Cox proportional model with forward selection, with *P* < 0.15 indicating inclusion and removal for variable selection. Model I: variables without recurrence. Model II: recurrence as time-invariant variables. Model III: recurrence as time-varying variables

In the final model, the clinical weight based on regression coefficients for each risk factor was 0.44 for older age, 0.68 for CSPH, 1.89 for early recurrence and 1.32 for late recurrence. The predicted risk score based on clinical weight combined with risk factors was:

$$ \mathrm{Risk}\ \mathrm{score}=\left(0.44\times \mathrm{older}\ \mathrm{age}\right)+\left(0.68\times \mathrm{CSPH}\right)+\left(1.89\times \mathrm{early}\ \mathrm{recurrence}\right)+\left(1.32\times \mathrm{late}\ \mathrm{recurrence}\right) $$

**Fig. 2:**
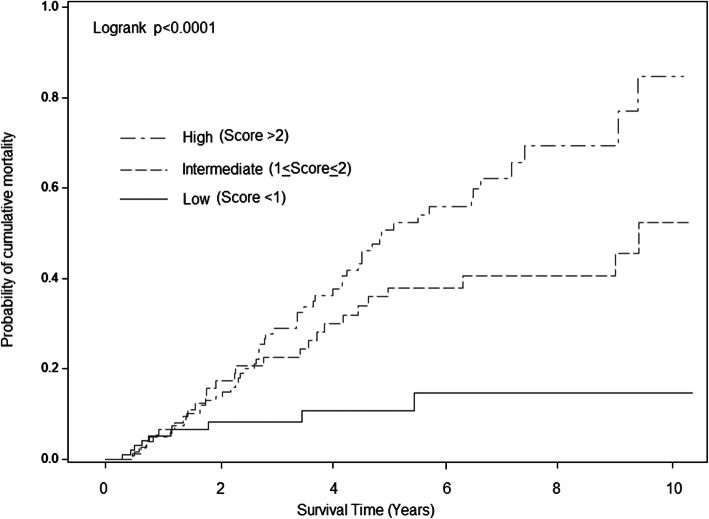
Cumulative risk for mortality in early-stage HCC patients after primary curative treatment with low, intermediate, and high predicted scores in our cohort, scores of < 1, 1 to 2, and > 2 indicate low, intermediate, and high risk, respectively

After we excluded those who did not complete follow-up, the area under ROC of the predicted risk score of 3- and 5-year OS was 77.9 and 76.8%, respectively. The optimal cut-off was score 1.71 and 1.74 for 3- and 5-year OS. A more recent independent cohort of early HCC at our institute was used for internal validation. The C-indexes for 3-year and 5-year OS were 0.79 and 0.74, respectively. By using the cutoff score of 1.71, the overall sensitivity and specificity for 3-year OS were 92.3 and 50.0%, respectively. Meanwhile, the sensitivity and specificity for 5-year OS showed 90.0 and 63.2% respectively on the basis of the cutoff score of 1.74. We also categorized our cohort into the three risk groups with cutoff scores of 1 and 2 by using previous estimated clinical weight (Fig. 2). The difference of cumulative mortality among the risk categories was significant (*p* < 0.0001). The 3- and 5-year overall mortality were 8.3 and 10.8% respectively in the low-risk category; 22.6 and 38.0% respectively in the intermediate-risk category (HR = 2.71; 95% CI, 1.27–5.78); and 29.0 and 50.7% respectively in the high-risk category (HR = 4.50; 95% CI, 2.22–9.10).

### Subgroup analysis for HCC prognosis between surgical and nonsurgical methods

**Fig. 3:**
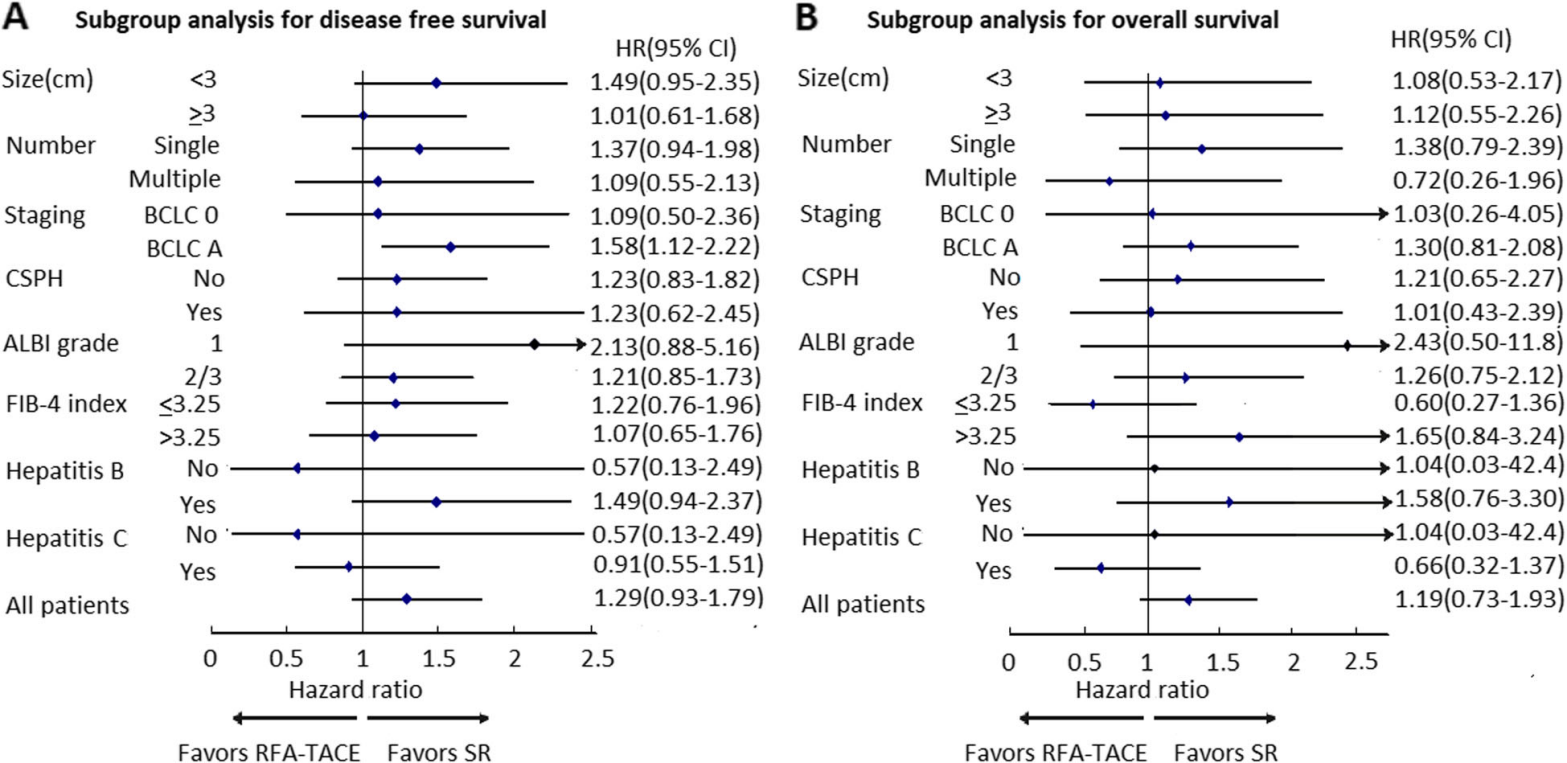
Adjusted RFA-TACE to SR ratios of hazard ratios and their 95% confidence intervals (horizontal lines) for the association of various liver reserve and tumor factors and long-term outcomes of HCC. **a** Subgroup analysis for disease-free survival. **b** Subgroup analysis for overall survival. All estimated results were based on Cox proportional hazard regression with adjustment for age, gender, tumor size, tumor number, portal hypertension, albumin level and etiologies of hepatitis

Figure 3 showed the relative risk of DFS and OS of HCC after curative treatment in associated with treatment modalities after adjusting for various clinical factors, stratified by various tumor and liver reserve status. Compared to SR, the HCC patients with tumor size < 3 cm, single tumor, albumin-bilirubin (ALBI) grade 1 and those without CSPH who received RFA-TACE treatment had higher risks of recurrence in the univariate analysis (Supplementary Table S1). However, RFA-TACE was not significantly associated with DFS with addition of various clinical factors. Only those with BCLC stage A were beneficial from SR in terms of DFS (HR = 1.58, 95% CI = 1.12–2.22) (Fig. 3a). On the other hand, RFA-TACE and SR showed similar effectiveness in terms of OS in all the subgroups (Fig. 3b). Among those with BCLC stage A, the propensity score matching method with generation of 46 matching pairs also revealed the similar findings (Supplementary Figure S1).

## Discussion

Transcatheter arterial chemoembolization (TACE) and radiofrequency ablation (RFA) are the mainstay of nonsurgical methods for early HCC and have provided minimally invasive options that may individually or in combination yield successful HCC eradication with maximal maintenance of liver reserve. In Japan, a large number of physicians have employed TACE before RFA treatment for 3 cm or larger HCC in order to minimize the likelihood of occult microsatellite lesions and microvascular invasion [16]. A meta-analysis based on eight randomized controlled trials also revealed that combined TACE and RFA yielded better survival benefits than RFA monotherapy for patients with intermediate-sized HCC (3 cm < tumor size < 5 cm) [17]. There is some evidence supporting the synergic effects. Firstly, occlusion of the hepatic arterial flow by embolization reduces the cooling effect of hepatic blood flow on thermal coagulation. Secondly, the iodized oil and gelatin sponge particles used in TACE fill the peripheral portal vein around the tumor by arterioportal shunting, thus compromising microscopic tumor spread [8, 18]. Thirdly, retained oil within HCC after TACE can also be helpful for targeting undetected liver tumors.

In our study, adjustment by propensity score matching indicated that the patients who underwent RFA-TACE had similar OS and DFS rates as the patients who underwent SR. Yamakado et al. first compared the efficacy of RFA combined with TACE to that of SR in early stage HCC patients with Child-Pugh class A liver profiles [19]. The 5-year DFS and OS rates of the two groups were similar, a finding which was in accordance with the results of our study. Meanwhile, the 5-year OS rates of our cohort (61% in the RFA-TACE group and 65% in the SR group) were also similar to that of patients with early HCC who underwent RFA-TACE or SR, which were as high as 58–75% and 61–81%, respectively [19–21]. Although more and more research demonstrated the superiority of SR compared to nonsurgical methods in the management of early HCC, the generalization was still limited by the certain sample characteristics such as heterogeneous baseline characteristics, relatively small sizes, and lack of sampling beyond a single institution [21, 22]. Due to ethical issues, it is difficult to establish a well-designed randomized trial to compare the effectiveness of nonsurgical treatment with that of SR. Our propensity score model balanced the baseline characteristics of the treatment groups and thus may have provided a suboptimal comparison between the two groups.

To rule out the possibility of treatment discrepancy after HCC recurrence, we performed a sensitivity test by comparing the post-recurrence survival rates between the two groups. A similar survival rate with no statistical difference after recurrence (*P* = 0.43) may imply that the recurrence and mortality outcomes in our study are largely attributable to the effects of initial treatment modalities.

In our study, both treatment groups had low rates of major complications, and there were no treatment-related deaths. It is well known that SR can be safely done in well selected patients with moderate portal hypertension, and a preoperative assessment that considers the planned extension of resection and the score of Model for End-stage Liver Disease (MELD) can adequately stratify the risk of postoperative liver failure [23]. In our SR cohort, minor hepatectomy (less than 2 segments) was performed in more than 88% (23/26) patients with clinically significant portal hypertension (CSPH). We believe a limited curative resection could still be planned in those with CSPH, and this finding was in accordance with the results of previous studies [24, 25]. Meanwhile, RFA and TACE can also be performed in well-selected Child-Pugh B patients with very good outcome [26, 27]. However, our cohort included only a limited number of Child-Pugh B patients and 82% (14/17) of them received RFA-TACE. This finding suggests that patients with moderate liver reserve were more likely to receive nonsurgical methods, but the generalization of our results to those with intermediate cirrhosis was limited due to the small sample size. Nevertheless, the rate of major complications related to RFA-TACE in our study was 1.1%, which was inferior to the rate of SR and was comparable to previous reports which showed a range between 0 and 3.7% [17, 19, 20]. Since the nonsurgical treatment is much less invasive and is associated with shorter hospital stays, the results suggest that RFA-TACE could serve as a safer and less costly alternative form of early HCC treatment.

In the present study, both multinodularity and elevated AFP level were tumor factors significantly associated with early recurrence. These findings suggest that dissemination of the primary tumor via microsatellite lesions and microvascular invasion was attributed to early recurrence [28, 29]. In addition, multinodularity also appeared to be associated with late recurrence, resulting from the “field effect” related multicentric metachronous tumors [30]. On the other hand, factors associated with late recurrence, such as chronic hepatitis B and chronic hepatitis C, are thought to be variables reflecting increased carcinogenicity of the background liver [31, 32]. The long-term effect of viral eradication on HCC recurrence is another interesting issue for clinical physicians [33]. Since this study enrolled patients from January 2003 to December 2016, nucleoside analogs (NA) and pegylated interferon plus ribavirin (PR) were available regimens for antiviral therapies in our native country. Due to the reimbursement for NA for HBV infection is strictly limited to specific indications in Taiwan, not included HCC and PR based treatment was relatively contraindicated in HCV-related HCC, few of our patients received antiviral therapies after curative HCC treatment [34]. With the launch of interferon-free regimens for hepatitis C treatment, an early intervention with direct-acting antivirals (DAA) was expected to improve the tumor cascade of liver parenchyma, thus reducing the risk of HCC recurrence [35, 36].

Our finding showed that the presence of early and late recurrence was associated with a 6.62-time and 3.75-time higher likelihood of mortality, respectively, compared with no recurrence. Intrahepatic metastasis and multicentric HCC development were previously thought to be the major mechanisms for early and late recurrence, respectively [9, 10]. Given the great influence of recurrence, it is important to consider treatment selection based on recurrence types: intrahepatic metastasis could be beneficial to targeted therapy based on the molecular profiles of the original tumor, and multicentric occurrence might be prevented by managing underlying liver disease. In addition, CSPH and older age also significantly contributed to overall survival rates. Studies from various countries have documented a positive association between portal hypertension and the risk of forming liver decompensation, and thus the increased risk of mortality as well [37, 38]. Similarly, the results of the present study demonstrated that CSPH was associated with increases in mortality of 1.97–fold. More and more evidence has suggested that antiviral therapies could reduce hepatic venous pressure gradient, but these findings were limited to those with earlier stage of liver cirrhosis [39, 40]. The presence of CSPH in patients with early-stage HCC may imply the consideration of liver transplantation before recurrence.

In the subgroup of BCLC stage A, our patients who underwent RFA-TACE had a similar OS but poorer DFS rates when compared with patients who underwent SR. The difference between DFS rates may be mainly due to local tumor progression. Nearly 65 % of our SR patients received anatomical segmentectomy. The advantage of complete resection of tumor tissue and portal territory containing the tumor may result in lower frequency of local tumor recurrence [41, 42]. The equivalent OS between RFA-TACE and SR may contribute to the higher repeatability of RFA-TACE procedures in the nonsurgical group for recurrence control. Meanwhile, many physicians had accepted that patients with good liver reserve should undergo SR while patients with poor liver reserve should receive nonsurgical therapy [43, 44]. In the present study, SR seemed to endow more advantages related to liver reserve in the univariate analysis, but the effect did not hold up once we adjusted for other clinical factors. Regarding to the long-term outcomes of early HCC, further research to assess the interaction between liver reserve and treatment modalities may be necessary.

This study has several strengths. Firstly, various pre- and post-operative factors in the past years have been used to predict the outcomes after curative early HCC treatment. The addition of recurrence information in our model not only quantifies the influence of both early and late recurrence but also increases the predictive power. Secondly, a female receiving SR for early HCC, for example, who may undergo recurrence within 1 year. Recurrence at either 3 or 12 months is couched under the same label (early recurrence) in a time-fixed model but may imply different prognosis for clinical physicians. By using recurrence as time-varying covariates, our new model accounts for the nature of the data better than a time-invariant one. Thirdly, previous reports only focused on the association between putative risk factors and outcomes after curative treatment. Our validated risk score with fair accuracy will be very helpful for general practitioners to identify patients prone to mortality for whom different management strategies may be indicated. Fourthly, risk factors associated with late recurrence were previously based on those who did not develop recurrence, hence subjects with early recurrence were abandoned in the analyses. A polytomous logistic regression model can simultaneously assess the risk factors of multiple outcome categories on the basis of a correct covariance matrix.

The present study also has some limitations. First, this study used a retrospective approach and a nonrandomized design; the choice of surgical or nonsurgical method was primarily based on the physician’s judgement and recommendation for a substantial number of patients with early HCC. Therefore, the introduction of selection bias was unavoidable. Second, we investigated patients in an endemic area of viral hepatitis. With the increasing evidence that antiviral therapies reduced recurrence after curative HCC treatment, further studies consisted of viral variables as well as treatment response may be warranted [45, 46]. Third, although we used the propensity score method to minimize the selection bias when comparing the survival rates of the patients who underwent RFA-TACE to those of the patients who underwent SR, the sample size in the propensity analysis was reduced and thus may have influenced the statistical power of the survival analysis. Fourth, since the duration of inclusion was long, many techniques and devices of HCC treatment have improved over time. The introduction of a clustered electrode RFA technique and drug-eluting bead TACE, for example, has offered outcomes with improved consistency and replicability [47, 48]. However, only a limited number of the patients included in this study were treated with these techniques. The influence of these new techniques on our result was minimal but should be further assessed. Finally, our predictive model was based on Taiwanese residents in a single institute. Since the etiologies of HCC varied among different races, the predicted score needs to be validated in the near future before generalization to western populations.

In conclusion, our propensity score model provides evidence that, in comparison to SR, RFA with or without chemoembolization can result in comparable long-term overall survival for early HCC patients without increased safety concerns. However, SR yields better DFS rates than nonsurgical methods in the BCLC stage A subgroup. Our models, based on recurrence types, are clinical relevant and predict long-term survival with fair accuracy. Further prospective studies that explore the external validity and applicability of our model are still required.

## Supplementary Information

**Additional file 1: Table S1.** Overall and tumor and liver reserve-specific relative hazards of disease-free survival and overall survival of HCC after curative treatment in association with treatment modalities with SR as reference strategy. **Figure S1.** Comparison of survival curves of the patients with HCC in Barcelona Clinic Liver Cancer (BCLC)  A stage who underwent RFA-TACE or SR.(A) Cumulative OS curves of patients with HCC in BCLC A stage who underwent RFA-TACE and patients who underwent SR. (B)Cumulative DFS curves of patients with HCC in BCLC A stage who underwent RFA-TACE and patients who underwent SR. (C) After propensity score matching, the cumulative OS curves of patients with HCC in BCLC A stage who underwent RFA-TACE and patients who underwent SR. (D) After propensity score matching, the cumulative DFS curves of patients with HCC in BCLC A stage who underwent RFA-TACE and patients who underwent SR.

## Data Availability

The datasets used and analyzed during the current study are available from the corresponding author on reasonable request.

## Abbreviations

HCC: Hepatocellular carcinoma
SR: Surgical resection
RFA: Percutaneous radiofrequency ablation
TACE: Transcatheter arterial chemoembolization
RFA-TACE: Percutaneous radiofrequency ablation with or without transcatheter arterial chemoembolization
OS: Overall survival
DFS: Disease-free survival
CSPH: Clinically significant portal hypertension
BCLC: Barcelona Clinic Liver Cancer staging system
EASL: European Association for the Study of the Liver
AASLD: American Association for the Study of Liver Diseases
ROC: Receiver operating characteristic
ALT: Alanine aminotransferase
AST: Aspartate aminotransferase
ALBI: Albumin-bilirubin
AFP: Alpha-fetoprotein
INR: International normalized ratio
HBV: Hepatitis B virus
HCV: Hepatitis C virus
aHR: adjusted hazard ratio
AIC: Akaike information criterion
DAA: Direct-acting antivirals
MELD: Model for End-stage Liver Disease
NA: Nucleoside analogs
PR: Pegylated interferon plus ribavirin
