# Supplementary material for: Factors predicting long-term outcomes of early-stage hepatocellular carcinoma after primary curative treatment: the role of surgical or nonsurgical methods
Source: BMC Cancer. 2021 Mar 8;21:250. doi: 10.1186/s12885-021-07948-9 (PMC7941925; doi:10.1186/s12885-021-07948-9)
Supplement: Supplementary file 1 — Additional file 1: Table S1. Overall and tumor and liver reserve-specific relative hazards of disease-free survival and overall survival of HCC after curative treatment in association with treatment modalities with SR as reference strategy. Figure S1. Comparison of survival curves of the patients with HCC in Barcelona Clinic Liver Cancer (BCLC) A stage who underwent RFA-TACE or SR.(A) Cumulative OS curves of patients with HCC in BCLC A stage who underwent RFA-TACE and patients who underwent SR. (B)Cumulative DFS curves of patients with HCC in BCLC A stage who underwent RFA-TACE and patients who underwent SR. (C) After propensity score matching, the cumulative OS curves of patients with HCC in BCLC A stage who underwent RFA-TACE and patients who underwent SR. (D) After propensity score matching, the cumulative DFS curves of patients with HCC in BCLC A stage who underwent RFA-TACE and patients who underwent SR. [file 12885_2021_7948_MOESM1_ESM.pdf]

Supporting information

Kuo MJ, Mo LR, Chen CL. Factors predicting long-term outcomes of early-stage hepatocellular carcinoma after primary curative treatment: The role of surgical or nonsurgical methods

Table S1 Overall and tumor and liver reserve-specific relative hazards of disease-free survival and overall survival of HCC after curative treatment in association with treatment modalities with SR as reference strategy

Figure S1 Comparison of survival curves of the patients with HCC in Barcelona Clinic Liver Cancer (BCLC) stage A HCC who underwent RFA-TACE or SR. (A) Cumulative OS curves of patients with HCC in BCLC A stage who underwent RFA-TACE and patients who underwent SR. (B) Cumulative DFS curves of patients with HCC in BCLC A stage who underwent RFA-TACE and patients who underwent SR. (C) After propensity score matching, the cumulative OS curves of patients with HCC in BCLC A stage who underwent RFA-TACE and patients who underwent SR. (D) After propensity score matching, the cumulative DFS curves of patients with HCC in BCLC A stage who underwent RFA-TACE and patients who underwent SR.

Table S1 Overall and tumor and liver reserve- specific relative hazards of disease-free survival and overall survival of HCC after curative treatment in association with treatment modalities with SR as reference strategy

	No. of patients	Disease-free survival		Overall survival	
Size and number	RFA-TACE/SR	Crude HR (95% CI)	Adjusted HR* (95% CI)	Crude HR (95% CI)	Adjusted HR* (95% CI)
Size					
<3 cm	129/50	1.75(1.14-2.68)	1.49(0.95-2.35)	1.20(0.64-2.25)	1.08(0.53-2.17)
≥3cm	47/75	1.35(0.84-2.17)	1.01(0.61-1.68)	1.39(0.73-2.65)	1.12(0.55-2.26)
Number					
Single	126/104	1.50(1.06-2.13)	1.37(0.94-1.98)	1.40(0.85-2.31)	1.38(0.79-2.39)
Multinodulaity	50/21	1.29(0.70-2.37)	1.09(0.55-2.13)	0.74(0.31-1.76)	0.72(0.26-1.96)
Staging					
BCLC 0	47/18	1.12(0.54-2.30)	1.09(0.50-2.36)	1.12(0.30-4.21)	1.03(0.26-4.05)
BCLC A	129/107	1.77(1.27-2.46)	1.58(1.12-2.22)	1.37(0.88-2.15)	1.30(0.81-2.08)
CSPH					
No	131/99	1.61(1.13-2.29)	1.23(0.83-1.82)	1.27(0.75-2.16)	1.21(0.65-2.27)
Yes	45/26	1.31(0.74-2.32)	1.23(0.62-2.45)	1.09(0.53-2.22)	1.01(0.43-2.39)
ALBI grade					
1	35/22	2.49(1.19-5.22)	2.13(0.88-5.16)	2.33(0.62-8.81)	2.43(0.50-11.8)
2/3	141/101	1.41(1.02-1.95)	1.21(0.85-1.73)	1.16(0.74-1.81)	1.26(0.75-2.12)
FIB-4 index					
≤3.25	88/77	0.80(0.41-1.56)	1.22(0.76-1.96)	1.60(1.03-2.49)	0.60(0.27-1.36)
>3.25	88/48	1.39(0.91-2.14)	1.07(0.65-1.76)	1.47(0.82-2.63)	1.65(0.84-3.24)
HBV hepatitis					
No	17/21	1.41(0.50-3.96)	0.57(0.13-2.49)	0.98(0.18-5.35)	1.04(0.03-42.4)
Yes	70/65	1.54(0.99-2.39)	1.49(0.94-2.37)	1.38(0.71-2.69)	1.58(0.76-3.30)
HCV hepatitis					
No	17/21	1.41(0.50-3.96)	0.57(0.13-2.49)	0.98(0.18-5.35)	1.04(0.03-42.4)
Yes	82/36	1.08(0.68-1.71)	0.91(0.55-1.51)	0.76(0.40-1.43)	0.66(0.32-1.37)
Overall	176/125	1.57(1.17-2.11)	1.29(0.93-1.79)	1.25(0.82-1.91)	1.19(0.73-1.93)

*Based on Cox proportional hazard regression with adjustment for age, gender, tumor size, tumor number, portal hypertension, albumin level and etiologies of hepatitis.

CSPH: clinically significant portal hypertension; ALBI:albumin-bilirubin; HBV, hepatitis B virus; HCV, hepatitis C virus; BCLC, Barcelona-Clınic Liver Cancer staging system; RFA-TACE: Radiofrequency ablation with or without transcatheter arterial chemoembolization; SR: surgical resection.

Figure S1.

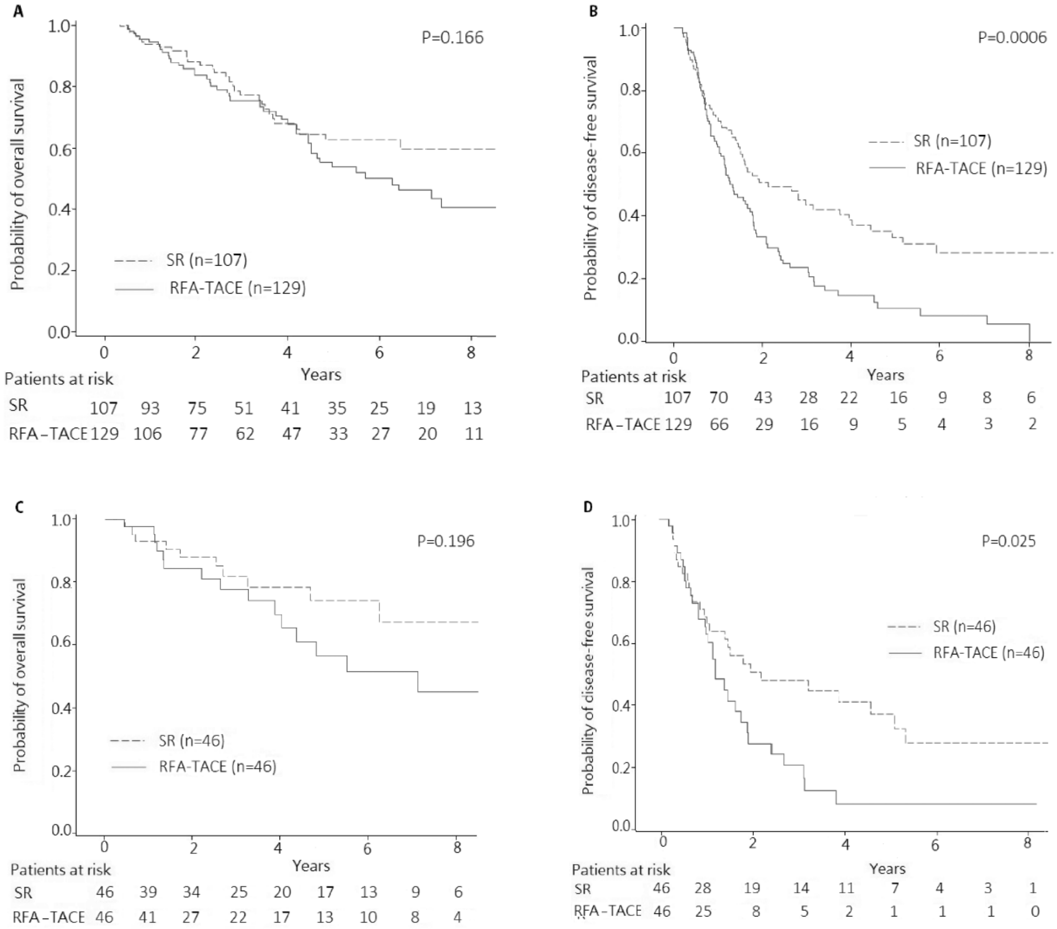
A
P=0.166
Probability of overall survival
SR (n=107)
RFA-TACE (n=129)
Years
Patients at risk
SR 107 93 75 51 41 35 25 19 13
RFA-TACE 129 106 77 62 47 33 27 20 11
B
P=0.0006
Probability of disease-free survival
SR (n=107)
RFA-TACE (n=129)
Years
Patients at risk
SR 107 70 43 28 22 16 9 8 6
RFA-TACE 129 66 29 16 9 5 4 3 2
C
P=0.196
Probability of overall survival
SR (n=46)
RFA-TACE (n=46)
Years
Patients at risk
SR 46 39 34 25 20 17 13 9 6
RFA-TACE 46 41 27 22 17 13 10 8 4
D
P=0.025
Probability of disease-free survival
SR (n=46)
RFA-TACE (n=46)
Years
Patients at risk
SR 46 28 19 14 11 7 4 3 1
RFA-TACE 46 25 8 5 2 1 1 1 0
